# Taurocholic Acid and Glycocholic Acid Inhibit Inflammation and Activate Farnesoid X Receptor Expression in LPS-Stimulated Zebrafish and Macrophages

**DOI:** 10.3390/molecules28052005

**Published:** 2023-02-21

**Authors:** Xutao Ge, Shaoze Huang, Can Ren, Lu Zhao

**Affiliations:** 1 Pharmaceutical Informatics Institute, College of Pharmaceutical Sciences, Zhejiang University, 866 Yuhangtang Road, Xihu District, Hangzhou 310058, China; 2 Department of Medicine, Chiatai Qingchunbao Pharmaceutical Co., Ltd., Hangzhou 310023, China; 3 Department of Vascular Surgery, The Second Affiliated Hospital of Zhejiang University Medical School, Hangzhou 310007, China

**Keywords:** *Calculus bovis*, taurocholic acid, glycocholic acid, farnesoid X receptor, zebrafish

## Abstract

A hyperactive immune response can be observed in patients with bacterial or viral infection, which may lead to the overproduction of proinflammatory cytokines, or “cytokine storm”, and a poor clinical outcome. Extensive research efforts have been devoted to the discovery of effective immune modulators, yet the therapeutic options are still very limited. Here, we focused on the clinically indicated anti-inflammatory natural product *Calculus bovis* and its related patent drug Babaodan to investigate the major active molecules in the medicinal mixture. Combined with high-resolution mass spectrometry, transgenic zebrafish-based phenotypic screening, and mouse macrophage models, taurochiolic acid (TCA) and glycoholic acid (GCA) were identified as two naturally derived anti-inflammatory agents with high efficacy and safety. Both bile acids significantly inhibited the lipopolysaccharide-induced macrophage recruitment and the secretion of proinflammatory cytokines/chemokines in in vivo and in vitro models. Further studies identified strongly increased expression of the farnesoid X receptor at both the mRNA and protein levels upon the administration of TCA or GCA, which may be essential for mediating the anti-inflammatory effects of the two bile acids. In conclusion, we identified TCA and GCA as two major anti-inflammatory compounds in *Calculus bovis* and Babaodan, which could be important quality markers for the future development of *Calculus bovis*, as well as promising lead compounds in the treatment of overactive immune responses.

## 1. Introduction

Bacterial infection is one of the leading causes of death worldwide [1]. Upon inflammation, the innate immune system is activated, which rapidly synthesizes and releases various cytokines and chemokines, such as interleukin-6 (IL-6), tumor necrosis factor-α (TNF-α), and c-c motif chemokine ligand 2 (CCL-2), to further augment the inflammatory responses and recruit more innate immune cells to clear the invading pathogens [2,3]. However, dysregulated inflammation can be harmful [4]. During excessive inflammation, the uncontrolled release of proinflammatory cytokines will also damage normal cells [5]. In serious cases such as sepsis, the overactivated immune response can cause organ damage and even death [6,7]. Hyperactivated inflammatory responses and cytokine storm are also correlated with the poor outcome of the SARS-CoV-2 infection. It is proven that severe COVID-19 patients exhibit high levels of inflammatory cytokines and chemokines [8,9]. Current therapies for immune hyperactivation include glucocorticoids and non-steroidal anti-inflammatory drugs (NSAIDs); however, they are cytotoxic and may cause other diseases, including diabetes and osteoporosis [10,11]. Therefore, how to control the inflammatory response during infection remains an overwhelming challenge.

Medicinal herbs and animal-sourced natural products have been used to treat inflammation in multiple geographical regions around the world for centuries. The high diversity in chemical structure and bioactivity makes natural products an inviting source for the identification of new lead compounds [12,13]. However, due to the complexity of their chemical components, the active substances and pharmacological mechanisms of most natural products remain unclear, which severely impedes their further application. *Calculus bovis* (*C. bovis*) is the dried gallstone of cattle widely used in China, Japan, and many other Asian countries for the treatment of stroke, convulsions, epilepsy, and high fever [14]. *C. bovis* is also the main component of many patent drugs. Babaodan (BBD), for example, is a traditional Chinese formula, approved in 2020 by the National Medical Products Administration of China (Med-drug permit no. Z10940006), in the treatment of viral hepatitis, cholecystitis, angiocholitis, and urinary tract infection [15]. Despite long-term usage in clinical applications, the pharmacological mechanism of BBD remains unclear. Previous studies suggested the involvement of multiple signaling pathways in BBD-mediated anti-inflammatory responses, such as NLRP3 inflammasome, P13K/AKT/mTOR pathway, AMPK signaling, NF-κB, and MAPK signaling [15,16,17,18,19,20]. Although extensive work has been devoted to the study of *C. bovis* and its related natural drugs, the bioactive components in these medicinal mixtures remain elusive.

Zebrafish is a newly emerged vertebrate organism that has many advantages for chemical screening, such as small body size, high fertility, rapid development, larvae transparency, and low breeding cost. Besides, the innate immune system of zebrafish is highly similar to mammals. Most immune cells, inflammatory mediators, and receptors are evolutionarily conserved between zebrafish and mammals, which makes the zebrafish an appropriate model for the study of inflammation mechanisms [21]. Moreover, the availability of different transgenic lines carrying fluorescence-labeled cells in different organ systems further facilitated the in vivo imaging of zebrafish. For example, macrophage-labeled line *Tg(mpeg:eGFP)*, neutrophil-labeled line *Tg(lyz:DsRed)*, T-cell-labeled line *Tg(rag2:DsRed)* were all constructed in previous studies, and are suitable to study the endogenous distribution and migration of immune cells [22,23,24]. In our previous study, we used the transgenic lines *Tg(lyz:eGFP)* and *Tg(mpeg:eGFP)* to construct an inflammatory bowel diseases(IBD) model and a trauma model in zebrafish and successfully identified the active compounds in two herbal formulae, based on the endogenous imaging of immune cells [25,26].

Therefore, motivated by the high clinical significance of anti-inflammatory therapies and the potential efficacy of *C. bovis* in regulating the hyperactivated immune process, we propose that it is necessary to further investigate the anti-inflammatory effects and the active components in *C. bovis*. In this study, we combined both in vivo and in vitro models of lipopolysaccharide (LPS)-simulated bacterial infection, in transgenic zebrafish and mammalian cells, to systematically identify the active substances and potential mechanisms of BBD and *C. bovis*. The major molecular compositions of bile acids in *C. bovis* were analyzed by ultra-high-performance liquid chromatography/quadrupole time-of-flight mass spectrometry (UPLC-QTOF-MS), among which taurocholate acid (TCA) and glycocholic acid (GCA) were discovered as novel anti-inflammatory compounds.

## 2. Results

### 2.1. BBD Extract Attenuates LPS-Induced Zebrafish Inflammation

**Figure 1:**
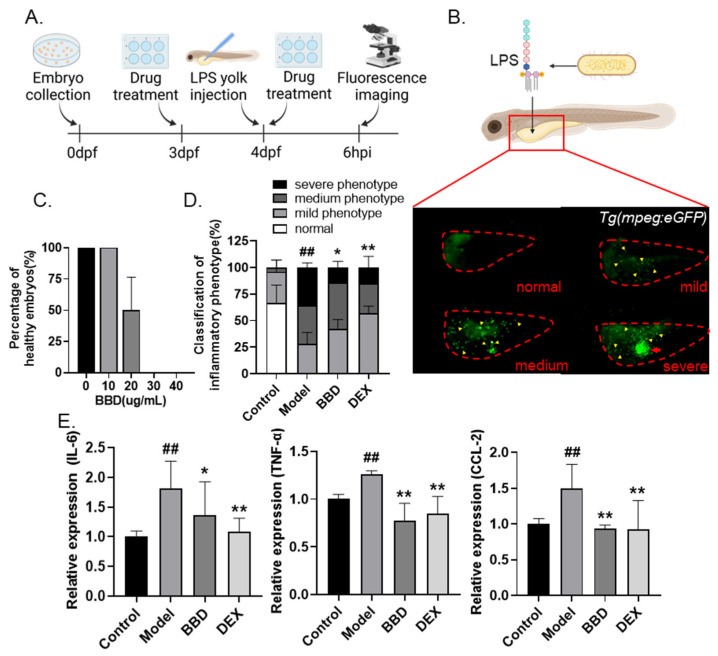
Anti-inflammatory effects of BBD on LPS-induced zebrafish inflammation model. (**A**) Flow chart of LPS-induced inflammation modeling. (**B**) Representative photos of zebrafish larvae of different degrees of inflammation. Embryos were classified as normal (no macrophage aggregation), mild (≤30 macrophages in yolk), medium (>30 macrophages but without an aggregation cluster), or severe (>30 macrophages and with an aggregation cluster). The red dotted line indicates zebrafish yolks, the yellow arrows indicate macrophages, and the red arrow indicates the aggregation cluster. (**C**) Toxicity of BBD treatment (n = 10). Larvae with cardiac edema, or body distortion, are defined as unhealthy. (**D**) The classification of inflammatory phenotypes in zebrafish embryos of different groups (n > 10). Control: PBS injection; model: LPS injection; DEX was used as positive control. The combined percentage of the medium and severe phenotypes was statistically analyzed among different groups. (**E**) The mRNA levels of IL-6, TNF-α, and CCL-2 in zebrafish embryos of different groups (n > 10). All the data are presented as the mean ± SEM of three independent experiments. # Compared with the control group; * compared with the model group; *, *p* < 0.05, ## or **, *p* < 0.01.

In order to examine the anti-inflammatory effects of BBD, we constructed an LPS-induced inflammation model in zebrafish as described previously [27]. Briefly, 4 days post-fertilization (dpf) zebrafish larvae were microinjected with LPS at the yolk sac to stimulate acute inflammation, and the severity of inflammation was evaluated by the endogenous imaging of macrophage accumulation in *Tg(mpeg:eGFP)* transgenic line (Figure 1A). Based on different levels of macrophage aggregation, embryos were classified as normal, mild, medium, and severe (Figure 1B).

Next, we evaluated the safety of BBD in zebrafish by evaluating the percentage of healthy embryos (alive embryos without observable developmental defects) after exposure to BBD at different doses from 3 dpf to 4 dpf. No toxic effect on the health of zebrafish embryos was observed for the 10 µg/mL BBD treatment, which concentration was selected for subsequent experiments (Figure 1C). After LPS microinjection, considerably increased macrophage aggregation was observed as compared with the PBS-injected control group, suggesting the successful stimulation of acute inflammation. Treatment with BBD or dexamethasone (DEX, as the positive control) was able to significantly inhibit the aggregation of macrophages (Figure 1D). Moreover, the transcriptional levels of pro-inflammatory cytokines IL-6 and TNF-α, as well as a macrophage chemokine CCL-2 were examined. The upregulated expression of all three biomarkers was greatly inhibited by BBD treatment (Figure 1E). Taken together, our results suggested that BBD has a strong anti-inflammatory effect in the LPS-induced inflammatory zebrafish model.

### 2.2. Calculus bovis Extract Attenuates LPS-Induced Zebrafish Yolk Sac Inflammation

**Figure 2:**
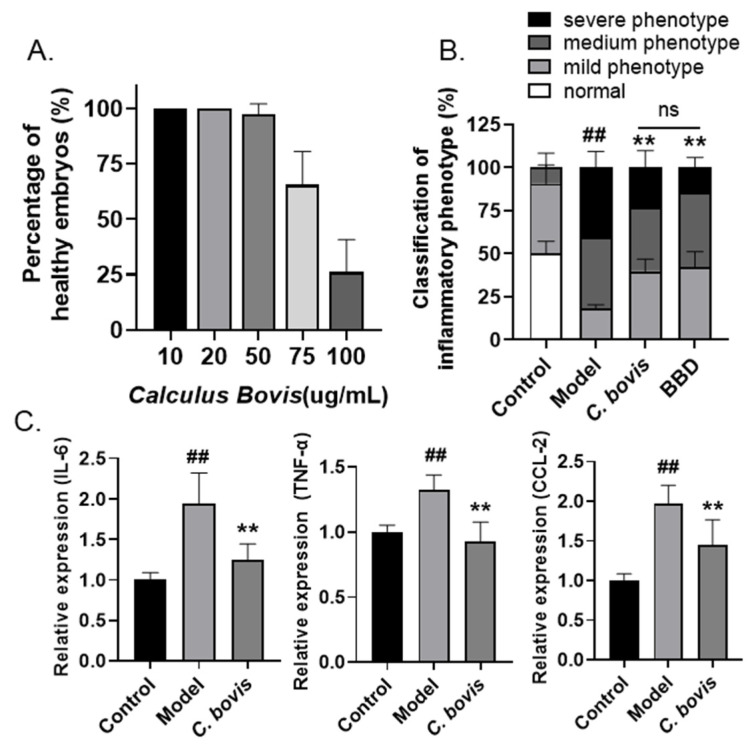
Anti-inflammatory effects of *C. bovis* on LPS-induced zebrafish inflammation model. (**A**) Toxicity of *C. bovis* treatment (n = 10). Larvae with cardiac edema, or body distortion, are defined as unhealthy. (**B**) The classification of inflammatory phenotypes in zebrafish embryos of different groups. The combined percentage of the medium and severe phenotypes was statistically analyzed among different groups (n > 10). Control: PBS injection; model: LPS injection. (**C**) The mRNA levels of IL-6, TNF-α, and CCL-2 in zebrafish embryos of different groups (n > 10). All the data are presented as the mean ± SEM of three independent experiments. # Compared with the control group; * compared with the model group; ## or **, *p* < 0.01.

As the dried gallstones of cattle, *C. bovis* has been used to treat fever, stroke, and other diseases in many geographical regions around the world for centuries [14], and is one of the major components in BBD. Therefore, we asked whether *C. bovis* extract is the major substance mediating the anti-inflammatory effects of BBD. Similarly, a toxicity assay was conducted to evaluate the safety of *C. bovis* extract in zebrafish embryos, and a concentration of 10 µg/mL was determined for the drug efficiency test (Figure 2A). Using the LPS-injected zebrafish inflammation model, significant downregulation of macrophage aggregation was also observed in embryos treated with *C. bovis* extract, reaching a similar level as the BBD-treated group regarding the combined percentage of medium and severe phenotypes (Figure 2B). Moreover, *C. bovis* also effectively reduced the expression of representative pro-inflammatory cytokines and chemokines (Figure 2C). Therefore, these results supported the hypothesis that *C. bovis* extract is the major anti-inflammatory component in BBD.

### 2.3. Identification of Chemical Constituents in C. bovis Extract

**Figure 3:**
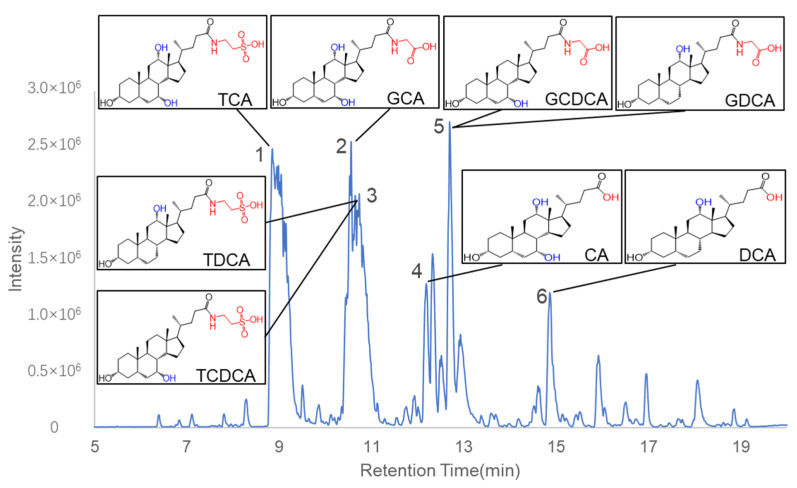
Base peak chromatogram of *C. bovis* in negative ion mode. Six peaks were identified as bile acids. The chemical structures of the eight major compounds chosen for the following studies were shown in boxes.

Next, we inquired what the major components are in *C. bovis* extract. A chemical profiling of *C. bovis* extract was performed by ultra-high-performance liquid chromatography/quadrupole time-of-flight mass spectrometry (UPLC-QTOF-MS) analysis. The base peak chromatogram of the *C. bovis* extract in negative ion mode is shown in Figure 3. We tried to identify the compounds in the six major ion peaks according to their *m*/*z*. Based on PeakView software 1.2, the structures of these compounds were further deduced by comparing their molecular formulae and fragment ions with those of existing substances from the literature and public databases (Table 1). Consistent with previous publications [28,29], bile acids were found to be the major components in the ion peaks. A list of eight major bile acids in *C. bovis* was selected for further pharmacological validation, including Taurodeoxycholate acid (TDCA), Deoxycholic acid (DCA), Taurocholic acid (TCA), Glycocholic acid (GCA), Taurochenodeoxycholic acid (TCDCA), Cholic acid (CA), Glycochenodeoxycholic acid (GCDCA), and Glycodeoxycholic acid (GDCA).

### 2.4. Screening of Anti-Inflammatory Bile Acids in Zebrafish Inflammatory Model

**Figure 4:**
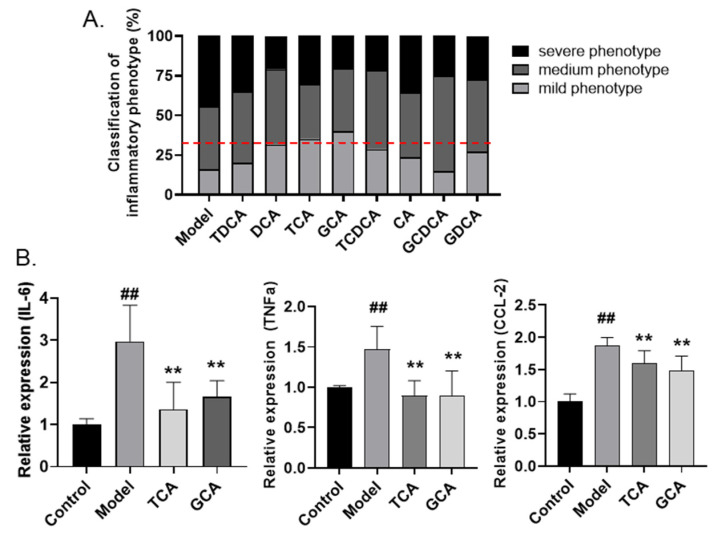
Screening active compounds of bile acids in zebrafish model. (**A**) The classification of inflammatory phenotypes in zebrafish embryos treated with different bile acids (n > 10). The cutoff line for positive hits is shown in red dotted line. (**B**) The mRNA levels of IL-6, TNF-α, and CCL-2 in zebrafish embryos of different groups (n > 10). Data are presented as the mean ± SEM of three independent experiments. # Compared with the control group; * compared with the model group; ## or **, *p* < 0.01.

Next, the anti-inflammatory effects of the eight bile acids in *C. bovis* extract were examined in the LPS-induced zebrafish inflammatory model. A rapid phenotypic screening was conducted by assessing the yolk aggregation of fluorescence-labeled macrophages, as described before. All the bile acids were administered at a concentration of 10 µg/mL. As shown in Figure 4A, most bile acids apparently alleviated the inflammation in the yolk sac, and we regarded those groups in which the combined percentage of medium and severe phenotypes is lower than 66.7% as positive hits. Among all the bile acids, TCA and GCA showed the strongest effects on reducing macrophage accumulation. We further examined the regulation of the two compounds on the expression of inflammatory-related biomarkers. Noticeably, the LPS-stimulated upregulation of IL-6, TNF-α, and CCL-2 were significantly inhibited by either TCA or GCA treatment (Figure 4B). Therefore, based on the zebrafish assay, we identified TCA and GCA as two major bile acids in the *C. bovis* extract exerting endogenous anti-inflammatory activities.

### 2.5. Validation of Anti-Inflammatory Bile Acids in LPS-Stimulated Macrophages

**Figure 5:**
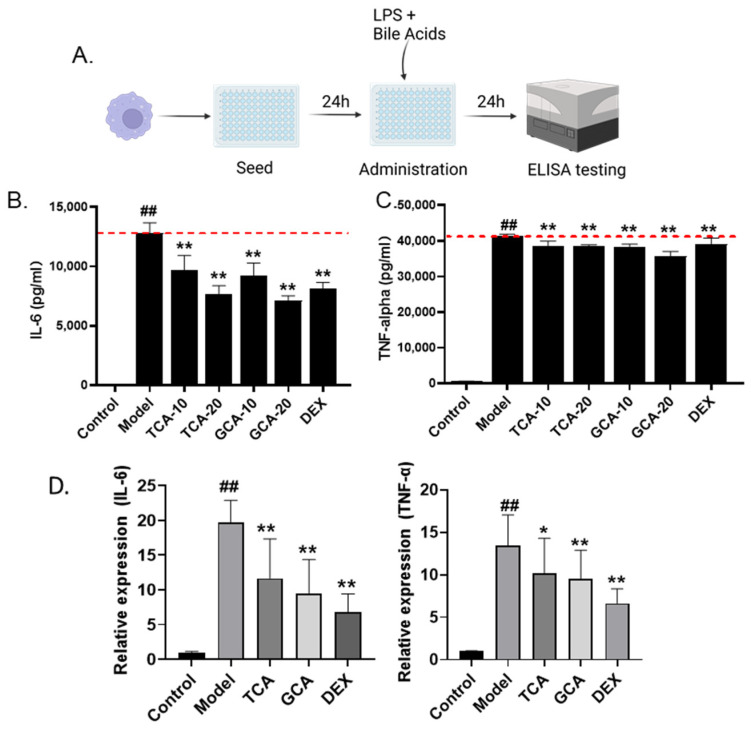
Validation of TCA and GCA in LPS-stimulated macrophages. (**A**) Flow chart of LPS-induced macrophage cell modeling. (**B**,**C**) The effect of TCA and GCA on the secretion of IL-6 and TNF-α in LPS-stimulated RAW264.7 cells. Each bile acid was tested at two concentrations of 10 and 20 µg/mL. DEX (8 µg/mL) is used as positive control. (**D**) Expression of inflammatory cytokines in TCA and GCA treated macrophages. All the data are presented as the mean ± SEM of three independent experiments. # Compared with the control group; *compared with the model group; *, *p* < 0.05, ## or **, *p* < 0.01.

In order to further validate the zebrafish screening result and analyze the inflammatory regulatory roles of TCA and GCA directly at the macrophage level, we further evaluated the anti-inflammatory effects of TCA and GCA on the LPS-stimulated mouse macrophage cell line RAW264.7 (Figure 5A). The supernatant was collected subsequently and the concentration of IL-6 and TNF-α was evaluated by the ELISA assay. Similar to the zebrafish analysis, LPS stimulation markedly increased the supernatant concentration of both IL-6 and TNF-α secreted by macrophages, which were significantly rescued by TCA or GCA (Figure 5B,C). The results of the QPCR assay further suggested their function in reducing the transcriptional expression of the two cytokines (Figure 5D). Thus, the anti-inflammatory roles of TCA and GCA were further validated in mouse macrophages.

### 2.6. TCA and GCA Increase the Expression of Farnesoid X Receptor

**Figure 6:**
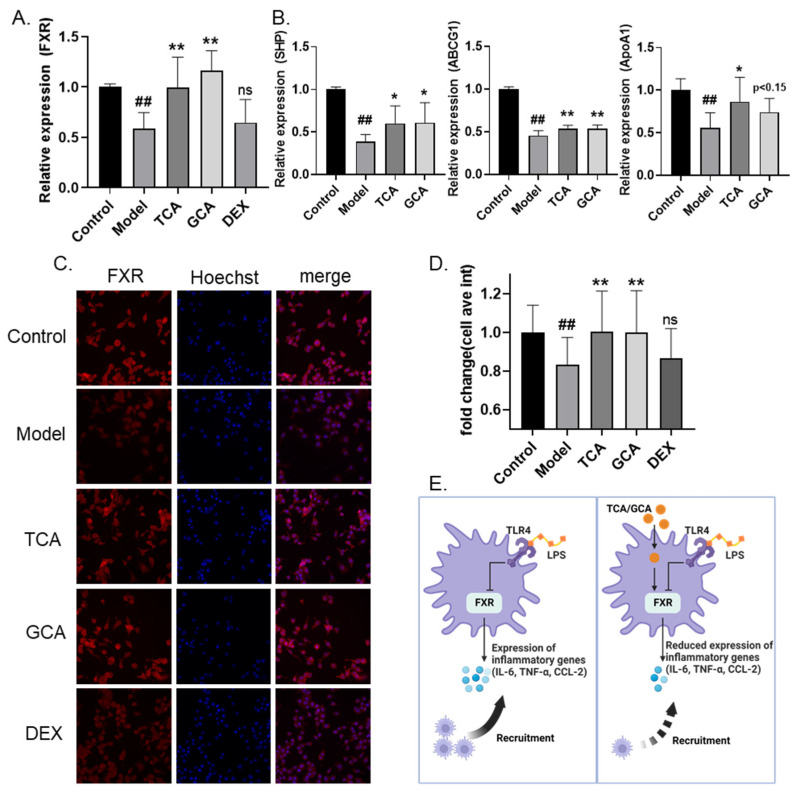
TCA and GCA increase the expression of FXR. (**A**) Increased FXR mRNA levels in bile acids-supplemented macrophage inflammation model. (**B**) Increased FXR downstream genes mRNA levels in bile acids-supplemented macrophage inflammation model. (**C**,**D**) Representative fluorescent images (**C**) and fluorescence quantification (**D**) of FXR immunofluorescence. (**E**) Schematic diagram of the proposed anti-inflammation mechanism of TCA and GCA. All the data are presented as the mean ± SEM of three independent experiments. # Compared with the control group; * compared with the model group; *, *p* < 0.05, ## or **, *p* < 0.01.

Previous studies suggested that the farnesoid X receptor (FXR) functions as a bile acid receptor and plays essential roles in inflammation inhibition [30,31]. Thus, we investigated the expression level of FXR in macrophages treated with TCA and GCA. Notably, both bile acids were able to greatly increase the transcriptional level of FXR (Figure 6A). In comparison, DEX, which is known to regulate inflammation through the glucocorticoid receptor-related pathway, shows no impact on FXR’s expression. Moreover, the mRNA levels of downstream factors in the FXR signaling, including short heterodimer partner (SHP), ATP-Binding Cassette Transporters G1(ABCG1), and apolipoprotein A1(ApoA1), were also significantly increased after treatment of either TCA or GCA, supporting the positive regulatory roles of both bile acids on the FXR signaling (Figure 6B). We further examined the impact of TCA and GCA on the protein expression of FXR by immunofluorescence. Similarly, TCA or GCA supplementation was able to effectively increase the protein level of FXR (Figure 6C,D). In addition, the impacts of both bile acids on another bile acid receptor, Takeda G protein-coupled receptor 5 (TGR5 or GPBAR1), were also tested. Interestingly, significant upregulation was detected for GCA on the transcriptional level of TGR5. A trend of increased expression of TGR5 was also observed after TCA, although with no statistical significance (Figure S1). Taken together, the above findings prove the positive regulation of TCA or GCA on the expression of bile acid receptors, especially FXR signaling, which may be essential for the anti-inflammatory activities of the two bile acids.

## 3. Discussion

Here, we identified TCA and GCA as two major bile acids in *C. bovis* and the TCM patent drug BBD with regard to their anti-inflammatory effects using both in vivo and in vitro models. TCA and GCA significantly inhibited macrophage migration and the cellular secretion of pro-inflammatory cytokines and chemokines, whose effects are possibly related to the upregulation of FXR expression (Figure 6D).

*C. bovis* is widely used in Asian countries such as China and Japan for the treatment of a variety of diseases, including high fever, convulsions, and stroke. Anti-inflammation has been suggested to be one of the major mechanisms mediating its broad activities by modern studies [14,32]. For example, a study in a formaldehyde-induced inflammatory pain model in rats suggested the protective effects of Taurine, a main component in *C. bovis*, via suppressing the activities of NF-κB and caspase-3 in brain tissue and downregulating inflammatory factors TNF-α and IL-1 [33]. Oral administration of *C. bovis* was also found to alleviate the inflammatory damage in the colon tissue of mice ulcerative colitis model induced by dextran sulfate sodium, accompanied by reduced levels of myeloperoxidase, SOD, and mRNA expression of IL-1β, IL-6, and TNF-α [34]. Nevertheless, natural *C. bovis* is rare and expensive. Only 0.05% of ox is estimated to have gallstones [14]. Moreover, as animal-derived natural products, the chemical components, and thus the bioactivity, may vary a lot. Due to the limited supplies of natural *C. bovis*, many substitutes are developed and used in some patent natural drugs, including *C. bovis* Sativus (in vitro cultured *C. bovis*) and *C. bovis* artifactus (artificially synthesized *C. bovis*). However, whether these substitutes can efficiently replace natural *C. bovis* in drug preparation is still debatable. To solve this problem, systematic deciphering of the active substances in *C. bovis* is of utmost importance.

The most important category of bioactive components in *C. bovis* is bile acids, with varying amounts in different types of *C. bovis* [35]. Under physiological conditions, bile acids are synthesized in the liver and secreted to the gastrointestinal tract to facilitate the organism’s digestion and absorption. Bile acids have established roles as signaling molecules in the metabolism and inflammation processes of obesity, type 2 diabetes, dyslipidemia, and nonalcoholic fatty liver disease [30,36,37]. The farnesoid X receptor (FXR; also known as NR1H4) is the first identified nuclear receptor of bile acids [38], which is mainly expressed in the liver, intestine, kidneys, adrenal glands, white adipose tissue, and immune cells [39]. FXR was suggested to downregulate the transcription of IL-6, probably by directly binding to IL-6’s promoter site [40]. A negative regulatory role of FXR on CCL-2 was also reported before, yet the mechanism is less clear [41,42]. Besides, FXR may also induce the expression of the short heterodimer partner (SHP), an atypical nuclear receptor that is increased during macrophage activation and has potential roles in inflammatory diseases [43]. Lack of SHP will lead to the activation of NF-κB and exacerbate hepatic inflammation and fibrosis in mice [44]. Aside from FXR, the ligand of G-protein-coupled receptors (GPCRs, such as TGR5) is also a crucial membrane receptor for bile acids. A recent study reported that through activation of the TGR5-cAMP-PKA axis, bile acids can trigger the phosphorylation and ubiquitination of NLRP3, thus alleviating NLRP3 inflammasome-dependent inflammation [45].

Although the pharmacological effects of *C. bovis* have been extensively studied, the major active components remain unclear. Here we identified TCA and GCA as the top-ranked bile acids with the strongest anti-inflammatory effect in *C. bovis*, based on both in vivo and in vitro models. According to the base peak chromatogram of *C. bovis* in our study, TCA and GCA have the shortest retention times compared to other identified bile acids, which is linked to higher polarity and water solubility. Compared with hydrophobic bile acids, it may be easier for hydrophilic bile acids to pass through the cell membrane and exert their intracellular anti-inflammatory effects. In fact, a previous study examined the content of bile acids in primary macrophages and proved that, although at a lower proportion in plasma [46], TCA and GCA are two bile acids that are enriched most in macrophages [47]. This may be an explanation for the observed strong effects of anti-inflammation and FXR signaling upregulation in our assay, despite the fact that the two bile acids were not among the strongest modulators of FXR [48].

In conclusion, we proposed an in vivo phenotypic screening model using a zebrafish model of acute inflammation induced by LPS microinjection. Combined with macrophage-labeled transgenic lines and fluorescence microscopy, we can quantify the accumulation of inflammation-recruited macrophages endogenously. We applied this model to the drug evaluation and screening of *C. bovis* and obtained the following major findings: Firstly, the anti-inflammatory effects of BBD and *C. bovis* were proven in the zebrafish model; Secondly, we identified eight bile acids in *C. bovis* via chemical analysis and screened for their anti-inflammatory activities in our model system. As a result, TCA and GCA were found to be two efficient anti-inflammatory compounds. Finally, based on an LPS-induced macrophage inflammation model, the anti-inflammatory effects of TCA and GCA were further verified and are possibly mediated through the upregulation of FXR signaling. Based on these findings, we propose that TCA and GCA could be important quality markers for the future development of *C. bovis* and its related patent drugs and serve as promising lead compounds in the treatment of bacterial infection. Nevertheless, some limitations still remain in our research. Firstly, the total amount of compounds of *C. bovis* included in our screening is still limited. More comprehensive screening for bioactive compounds in *C. bovis* is still needed in future studies. Quantitative analysis and multi-drug interaction assays are also necessary to better explain the anti-inflammatory function of *C. bovis*. Besides, we only examined the anti-inflammatory effects of TCA and GCA in the model of LPS-simulated bacterial infection. The potential roles of these two bile acids in other types of inflammation, such as viral infection, are warranted to be tested.

## 4. Materials and Methods

### 4.1. Sample Preparation

One gram of BBD (Xiamen Chinese Medicine Factory, 160730) was dissolved in 20 mL deionized water, ultrasonicated, and centrifugated at 2500 rpm for 5 min. Precipitate was dissolved in 20 mL of 70% ethanol, ultrasonicated, and centrifugated at 2500 rpm for 5 min. The total soluble solid in the 70% ethanol extract was determined and resolved in DMSO (SINOPHARM, 30072418) at a concentration of 5 mg/mL. *C. bovis* (Xiamen Chinese Medicine Factory) was extracted twice with a 10-fold volume of chloroform-methanol (1:1) for 1 h. The total soluble solid was obtained by centrifugation and concentration, followed by resolving in DMSO at a concentration of 20 mg/mL. Bile acids were dissolved in DMSO at a concentration of 10 mg/mL, including Taurodeoxycholate sodium salt (TDCA, DN0032), Deoxycholic acid (DCA, DQ0014), Taurocholic acid Sodium Salt (TCA, DN0030), Glycodeoxycholic acid(GDCA) purchased form Desite (Chengdu, China), Glycocholic acid (GCA, MB5234), Cholic acid (CA, MB5177) purchased form meilunbio (Dalian, China), Taurochenodeoxycholic acid (TCDCA, B20919), Glycochenodeoxycholic acid(GCDCA, B24724), dexamethasone (DEX, B25793) purchased from Yuanye Bio-Technology (Shanghai, China)

### 4.2. Zebrafish Husbandry and Animal Care Ethics

Wildtype AB strain and *Tg(mpeg:eGFP)* transgenic zebrafish were all obtained from the Laboratory Animal Center of Zhejiang University. Zebrafish were maintained following standard protocols. E3 medium (0.29 g/L NaCl, 0.013 g/L KCl, 0.048 g/L CaCl_2_·2H_2_O, 0.082 g/L MgCl_2_·6H_2_O, pH 7.2) was used as the zebrafish medium. Embryos were obtained through natural spawning. All zebrafish experiments were conducted according to the guidelines of the Animal Ethics Committee of the Laboratory Animal Center, Zhejiang University (No. 24609).

### 4.3. Zebrafish Acute Inflammation Model and Drug Administration

Three days post-fertilization (dpf), zebrafish were divided into control (PBS microinjection), model (LPS microinjection), and administration (LPS microinjection + drug administration) groups. Embryos in the administration groups were pretreated with BBD (10 µg/mL), *C. bovis* (10 µg/mL), bile acids (10 µg/mL), and dexamethasone (DEX, 20 µg/mL) (positive control), respectively. After 24 h of treatment, microinjection was performed in a 1 nL volume per larva with LPS (Solarbio LIFE SCIENCE, L8880) at a concentration of 2.5 mg/mL. The control group used phosphate-buffered saline (PBS) of the same volume as LPS. Embryos were retreated with drugs for 6 h right after recovery from anesthesia (0.02% tricaine). Wildtype AB strain embryos were used for the qPCR assay, and *Tg(mpeg:eGFP)* embryos were used for fluorescent phenotype analysis. At least 10 embryos were used for each group. Images were acquired under the Leica DMI 3000 B inverted microscope system (Leica Microsystems Inc., Morrisville, NC, USA).

### 4.4. UPLC-QTOF-MS Analysis

LC: Waters UPLC (Waters Corp., Milford, MA, USA), ACQUITY UPLC HSS T3 C18 column (1.7 μm, 2.1 × 150 mm; Waters Corp.) was used in the chromatographic experiments. The mobile phases were 0.1% formic acid-water (A) and 0.1% formic acid-acetonitrile (B). The linear gradient programs were as follows: 0/5, 10/50, 20/95, and 22/95 (min/B%); sample injection volume, 3 μL; column oven temperature, 50 °C; flow rate, 0.3 mL min^−1^; and the UV detector was set at 254 nm.

Mass spectrometry: AB TripleTOF 5600 Plus System (AB SCIEX, Framingham, USA) was used in the experiment. The optimal MS conditions are negative ion mode: a source voltage of −4.5 kV, and a source temperature of 550 °C. The pressures of Gas 1 (air) and Gas 2 (air) were set at 50 psi. The pressure of curtain gas (N2) was set to 35 psi. The maximum allowed error was set to ±5 ppm. Declustering potential (DP), 100 V; collision energy (CE), 10 eV. For MS/MS acquisition mode, the parameters were almost the same except that the collision energy (CE) was set at ±40 ± 20 eV, ion release delay (IRD) at 67, and the ion release width (IRW) at 25. In a full scan cycle of 1 s, the IDA-based auto-MS2 was performed on the eight most intense metabolite ions. The m/z scan ranges were set at 100–1500 Da for precursor ions and 50–1500 Da for product ions, respectively. The exact mass calibration was performed automatically before each analysis employing the Automated Calibration Delivery System.

### 4.5. Cell Culture and Drug Administration

Mice macrophage RAW264.7 cells (ATCC: TIB-71) were purchased from the cell bank of the Chinese Academy of Sciences (Shanghai, China). Cells were cultured in high-glucose Dulbecco’s Modified Eagle’s Medium (Gibco) supplemented with 10% heat-inactivated fetal bovine serum (CORNING, 35-076-CV) and 1% Penicillin-Streptomycin-Amphotericin B Solution (Beyotime, C0224) at 37 °C with 5% CO_2_. Cells were seeded to a 96-well plate (5000 cells/well) for 24 h. Wells were divided into control, model, and administration groups. The control groups were cultured with standard cell culture medium, the model group was treated with LPS (500 ng/mL), and the administration groups were simultaneously treated with LPS (500 ng/mL) and drugs for 24 h.

### 4.6. qPCR

Total RNA extraction was conducted using the RNA-Quick Purification Kit (RN001, ES Science) and quantified by nanodrop. Briefly, 2 µg of total RNA was reverse-transcribed into cDNA using a high-performance reverse transcription kit (Easy-Do, Zhejiang, China) according to the manufacturer’s protocol. Quantitative real-time PCR (qPCR) was performed using the UltraSYBR One-Step RT-qPCR Kit (CWBIO) according to the manufacturer’s protocol. Internal standardization was conducted prior to the gene expression of the control group used as a reference, and the 2^−∆∆Ct^ method was utilized for relative quantitative analysis. The primers used for qPCR are listed in Supporting Table S1.

### 4.7. ELISA Assay

The concentrations of TNF-α and IL-6 were quantified using ELISA kits which were purchased from eBioscience (San Diego, CA, USA) according to the manufacturer’s instructions.

### 4.8. Immunofluorescence

Cells were fixed in 4% paraformaldehyde at room temperature for 0.5 h and permeated by 2% Triton X-100 for 10 min. After 30 min of blocking with PBST (1%BSA and 22.52 mg/mL glycine), FXR primary antibody (1:100) (Proteintech, 25055-1-AP) was added to incubate overnight at 4 °C, followed by adding fluorescent secondary antibody TRITC-conjugated Goat Anti-Rabbit IgG(H + L) (Proteintech, SA00007-2) for 1 h. Then, Hoechst (1:1000) was added and incubated for 10 min to stain the cellular nucleus. Images were taken and analyzed by ImageXpress Micro Confocal (Molecular Devices, San Jose, CA, USA).

### 4.9. Statistical Analysis

All data are presented as the mean ± the standard error of the mean (SEM). Differences between the two groups were analyzed using the two-tailed Student’s *t*-test. Multiple group comparison was conducted by one-way ANOVA. A *p*-value < 0.05 was considered statistically significant.

## Figures and Tables

**Table 1:** Bile acids identified by UPLC-QTOF-MS in negative ion mode.

NO.	RT (min)	Molecular Formula	MW	Error (ppm)	Identified Compound	Fragment Ion
1	8.797	C_26_H_45_NO_7_S	515.29113	5.3	Taurocholic acid	514.2844[M-H]−
2	10.504	C_26_H_43_NO_6_	465.30849	2.7	Glycocholic acid	464.3030[M-H]− 446.2935[M-H-H_2_O]− 420.3130[M-H-CO_2_]− 402.3013[M-H-H_2_CO_3_]−
3	10.962	C_26_H_45_NO_6_S	499.29621	4	Tauroursodeoxycholic acid/Taurochenodeoxycholic acid/Taurodeoxycholic acid/Taurohyodeoxycholic acid	498.2915[M-H]−
4	12.133	C_24_H_40_O_5_	408.28703	−0.5	Cholic acid	407.2801[M-H]− 389.2695[M-H-H_2_O]− 345.2795[M-H-H_2_CO_3_]−
5	12.312	C_26_H_43_NO_5_	449.31358	4.8	Glycoursodeoxycholic acid/Glycodeoxycholic acid/Glycohyodeoxycholic acid/Glycochenodeoxycholic acid	448.3090[M-H]− 404.3194[M-H-CO_2_]− 386.3077[M-H-H_2_CO_3_]−
6	14.808	C_24_H_40_O_4_	392.29211	−1	Deoxycholic acid/Ursodeoxycholic acid/Chenodeoxycholic acid/Hyodeoxycholic Acid	391.2850[M-H]− 347.2952[M-HMCO_2_]− 327.2699[M-H-H_2_CO_3_]−

## Data Availability

Not applicable.

## Supplementary Materials

The following supporting information can be downloaded at: https://www.mdpi.com/article/10.3390/molecules28052005/s1, Figure S1: The mRNA levels of TGR5 in bile acids-supplemented macrophage inflammation model; Table S1: Primers used in this study.
