# Supplementary material for: Taurocholic Acid and Glycocholic Acid Inhibit Inflammation and Activate Farnesoid X Receptor Expression in LPS-Stimulated Zebrafish and Macrophages
Source: Molecules. 2023 Feb 21;28(5):2005. doi: 10.3390/molecules28052005 (PMC10003765; doi:10.3390/molecules28052005)
Supplement: Supplementary file 1 [file molecules-28-02005-s001.zip › molecules-2171011-supplementary.pdf]

Supplementary Materials for

Taurocholic Acid and Glycocholic Acid Inhibit Inflammation and Activate Farnesoid X Receptor Expression in LPS-Stimulated Zebrafish and Macrophages

Table S1. Primers used in this study.

Gene	Forward(5′-3′)	Reverse(3′-5′)
(zebrafish)eF-1α	AGAAGGCTGCCAAGACCAAG	AGAGGTTGGGAAGAACACGC
(zebrafish)il-6	CGCTAAGGCAACTGGAAGAC	CCAGACCACTGGGAAACACT
(zebrafish)tnf-α	ACCAGGCCTTTTCTTCAGGT	TTTGCCTCCGTAGGATTCAG
(zebrafish)ccl-2	GTCTGGTGCTCTTCGCTTTC	TGCAGAGAAGATGCGTCGTA
(mice)gapdh	AGGTCGGTGTGAACGGATTTG	GGGGTCGTTGATGGCAACA
(mice)il-6	CTGCAAGAGACTTCCATCCAG	GGGGTCGTTGATGGCAACA
(mice)tnf-α	CAGGCGGTGCCTATGTCTC	CGATCACCCCGAAGTTCAGTAG
(mice)fxr	GCTTGATGTGCTACAAAAGCTG	CGTGGTGATGGTTGAATGTCC

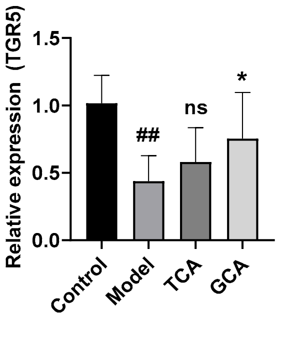

Figure S1. The mRNA levels of TGR5 in bile acids-supplemented macrophage inflammation model.
